# Training by Travel: Medical Education for OB/GYN Residents after *Dobbs*

**DOI:** 10.1017/amj.2025.10086

**Published:** 2025-12

**Authors:** Rachel Rebouché

**Affiliations:** G. Rollie White Chair in Law, The University of Texas School of Law, Austin, US

**Keywords:** Graduate medical education, abortion law, obstetrics, medicine, health care

## Abstract

After *Dobbs v. Jackson Women’s Health Organization*, the United States Supreme Court decision that overturned *Roe v. Wade* in 2022, OB/GYN residents’ access to abortion training, which is required in all accredited programs, has come under pressure. To receive the foundational training doctors in the field need, many residents in ban states travel to out-of-state programs where abortion is legal. But demand is high, travel for multiple weeks is expensive, and the capacity to train at host sites is limited.

Training by travel could have ripple effects for the quality of patient care. As the number of OB/GYNs continues to decrease, newly-trained providers will have different, and arguably diminished, skills in delivering not just elective but also medically necessary abortion care. And as exceptions for life and health become how legal, procedural terminations take place in one-third of the United States, there is no guarantee that the doctors in those states will feel comfortable providing that care.

This article explores the residency training provided today, providers’ and institutions’ navigation of abortion bans, and what changes in residency programs might mean for patient care in the coming years. Part I surveys the landscape of abortion law after *Dobbs*; even the strictest bans contemplate instances when abortion is medically required and legally permitted. Part II summarizes the pre- and post-*Dobbs* expectations for abortion training for OB/GYN residents, describing how graduate medical education has changed for residents in ban states. Part III assesses shorter and longer-term effects of a system that relies on travel and simulation (the use of models) or is out of compliance with national accreditation standards. Part IV concludes with potential paths forward that depend on state and national organizations supporting and funding the networks of health care professionals that facilitate training across the country.

“If you don’t even have the experience, the competency[,] and the comfort of doing [abortions] in planned settings, when … it’s two o’clock in the morning and you have someone who’s hemorrhaging at 18 weeks? I think that the future generations of our physicians are just not going to feel comfortable managing that on their own, and that’s going to be a detriment to patients beyond those who are seeking and needing abortion care.”^1^

## I. Introduction

Medical training for obstetrics and gynecology (OB/GYN) has shifted in significant ways. Shortly after *Dobbs v. Jackson Women’s Health Organization*,^2^ the United States Supreme Court decision that overturned *Roe v. Wade* ^3^ in 2022, news stories and medical and health journals documented the “brain drain” from OB/GYN residency programs in states with newly enacted or enforced abortion bans.^4^ Less discussed, however, has been how residency programs’ abortion training, required for accredited OB/GYN residencies, has come under pressure. To receive core family-planning education and clinical experience, many residents in ban states now travel to out-of-state training sites where abortion is lawful.^5^ But demand is great, travel for multiple weeks is expensive, and the clinical experiences at these sites can be limited by staff capacity.^6^ Residency programs also offer simulation — training with models and without seeing an actual patient — for miscarriage and abortion care.^7^

This shift suggests long-term effects for graduate medical education and the quality of patient care. For one, a cohort of the new generation of OB/GYNs will have limited experience treating patients with pregnancy loss or pregnancy complications that necessitate termination. As the number of OB/GYNs continues to decrease,^8^ newly-trained providers will have different, and arguably diminished, skills in providing medically necessary and emergency abortion care. And as life and health exceptions increasingly determine access to complicated care in many states, there is no guarantee that physicians practicing under bans will feel comfortable or sufficiently supported in delivering that care. Doctors in states that ban abortion may not only hesitate to perform abortions, because of criminal and civil liability, but also be unprepared to do so.^9^

This article focuses on changes to medical training in OB/GYN residency programs after *Dobbs.* In what follows, I demonstrate the relationship between the training provided today, providers’ navigation of abortion bans and exceptions, and what that might mean for patient care in the coming years. Part I surveys the landscape of abortion law after *Dobbs*; even the strictest abortion bans contemplate instances when abortion care is medically required and legally permitted. Part II summarizes the pre- and post-*Dobbs* expectations for training for OB/GYN residents, assessing how medical education has changed for residents in ban states. Part III contemplates shorter and longer-term effects of a system that relies on travel and simulation or is out of compliance with national accreditation standards. Part IV concludes with observations on potential paths forward that depend on state and national organizations supporting collaboration among the groups seeking to improve the current system.

## II. The Legal Landscape for Abortion Care

Post-*Dobbs* abortion law in the United States is marked by division and, in many places, there is a lack of clarity about how abortion laws apply.^10^ More than a dozen states enforce near-total bans^11^ and several others impose early gestational limits (including six-week and twelve-week bans).^12^ A number of states restrict abortion after viability.^13^ In some jurisdictions, the scope of restrictions has shifted due to ongoing litigation.^14^ Most state abortion bans impose both civil and criminal penalties for performing abortions, with some statutes authorizing severe sentences such as life imprisonment.^15^

Abortion bans include exceptions for life-threatening conditions and medical emergencies,^16^ and many are narrowly defined in physical terms only and expressly exclude psychological or emotional conditions.^17^ For example, some states prohibit a medical emergency exception from applying when the pregnant person threatens self-harm and when “based on a claim or diagnosis that the woman will engage in conduct which she intends to result in her death or in substantial and irreversible physical impairment of a major bodily function.”^18^

Fourteen state laws include exceptions for risks to health with language that varies,^19^ such as “a serious risk of substantial and irreversible physical impairment of a major bodily function”^20^ or “a condition that poses a serious risk of substantial and irreversible impairment of a major bodily function.”^21^ Some laws’ health exceptions fall outside these broad categories. For instance, Indiana’s exception allows an abortion in circumstances when the pregnancy “would pose a great risk of death or substantial physical impairment of the patient.”^22^ Mississippi’s ban permits for abortion if “a twenty-four-hour delay will create grave peril of immediate and irreversible loss of major bodily function.”^23^

Providers experience uncertainty in applying this language and thus difficulty in gauging when and if an exception has been met.^24^ News reports and court records document cases in which pregnant patients were denied abortion care despite significant medical need, resulting in severe injury and, in some cases, death.^25^ Maxine Eichner and her co-authors conducted in-depth, qualitative interviews with maternal fetal medicine physicians and described,

recurring gaps between the ways that physicians make abortion decisions in standard medical practice and the language of medical exceptions[, which] create uncertainty regarding whether, among other issues, the level of risk to life or health experienced by the patient rises to the threshold required by state law, and whether and when the patient’s medical risk meets the exception’s requirements regarding timing of the abortion.^26^

Health care professionals report misalignment between clinical judgment and statutory language, and a paucity of publicly available guidance from legislatures, courts, medical boards, and health departments.^27^ When state entities have issued guidance, the results are mixed. Following a decision by the Texas Supreme Court, the Texas Medical Board approved guidelines to clarify when doctors can legally intervene in a medical emergency and what they must document, although the rule was criticized as incomplete.^28^ Texas Senate Bill 31, enacted in August 2025, incorporates recent case law on the medical emergency exception and reasonable medical judgment, requiring one hour of continued medical education on Texas abortion law for physicians providing obstetric care.^29^

Doctors are not alone in grappling with state laws. Nadia Sawicki notes that some hospital administrators may prioritize minimizing legal exposure, and recent studies suggest that hospital attorneys would rather settle a malpractice claim than risk criminal investigation or attract the attention of the state legislature.^30^ The result is chill and confusion, or what one article deems “hesitant medicine” by institutions and lawyers.^31^ Of course, liability aversion is not uniformly true of actors in ban states. Yvette Lindgren and Michelle Oberman recently wrote about hospital personnel in Indiana who issued guidance that, though somewhat heavy in process, facilitates medical judgment and centralizes the process for approving abortions under the state law’s exceptions.^32^

The need for medically indicated abortions or urgent medical care continues regardless of state laws.^33^ Abortion is a treatment for people with pre-eclampsia, risk of hemorrhage, or severe pulmonary hypertension.^34^ The care for pregnancy loss and pregnancy complications is vital, no less so under the laws described here.^35^ Part II surveys the current state of medical training for physicians who are expected to respond to patients’ needs under abortion bans.

## III. Shifts in Medical Training for Abortion Care

Overturning *Roe* did not overturn the expectations of what physicians should learn. OB/GYNs are expected to provide comprehensive pregnancy options counseling and to competently manage early pregnancy loss and uterine evacuation, including abortion.^36^ This Part reviews the national standards mandating abortion training, before and after *Dobbs*, and how residency programs for OB/GYNs have responded to abortion bans. Residents can opt out of abortion training, and resident education always has been limited in various regions and at religiously affiliated health care facilities.^37^ But gaps in training availability are widening.

### A. Pre- and Post-*Dobbs* Training

This section primarily focuses on residency programs for OB/GYNs to illustrate how abortion training has occurred and occurs in the United States. This article does not intend to suggest that OB/GYNs are the only health care providers who deliver abortion services. Training in abortion care occurs in other specialties, such as in family medicine as well as in emergency and internal medicine.^38^ Twenty states permit advanced practice clinicians to provide abortion care.^39^ As set out in Part III, more training for a greater diversity of health care professionals is needed. But this section focuses on OB/GYNs in part because the Accreditation Council for Graduate Medical Education (ACGME), which governs the accreditation of residency programs, explicitly requires that OB/GYN residency programs provide access to training in abortion care.^40^ In addition, studies published before *Dobbs* reported that OB/GYNs provide over seventy percent of abortion care in the United States.^41^

Following *Dobbs*, the ACGME clarified that programs in legally-restrictive locations must continue to arrange training access in states where abortion is lawful.^42^ The ACGME mandates for all OB/GYN programs nationwide: (1) training, or access to training, in the provision of abortion; (2) an opt-out option for any resident with religious or moral objections; and (3) the provision of experience in managing complications of abortions and training in all forms of contraception.^43^ This mandate covers all accredited institutions, including public, private, urban and rural programs.^44^ However, federal law stipulates that health care entities cannot lose federal funding or accreditation status for failing to offer, provide, or make arrangements for training on induced abortion.^45^

Family planning training, including abortion, is a standard component of an OB/GYN residency, often delivered via a dedicated rotation or integrated curriculum.^46^ At Stanford Medicine, for example, family planning is integrated throughout the residency program.^47^ Stanford residents should acquire competency in performing first- and second-trimester abortions as well as assessing and managing pregnancy complications.^48^ Stanford offers third-year residents opportunities to train with community partners, including Planned Parenthood affiliates.^49^ The Swedish Medical Center in Seattle requires residents to learn, among various skills, how to provide contraceptive counseling, perform both first- and second-trimester abortions, and triage any complications that arise from outpatient terminations.^50^ This rotation additionally focuses on complicated counseling, office procedures, and the use of an operating room.^51^

Residency programs incorporate clinical training in rotations beyond family planning. Clinical training includes the provision of pelvic exams, administration of anesthesia, and patient education. For instance, NYU Langone Health requires residents to “take ownership, develop surgical skills, and improve clinical decision making”^52^ for “complex family planning” throughout all “research, clinical, and surgical” settings.^53^ The Midwest’s Corewell Health (Corewell), located in Michigan, requires residents to complete multiple rotations in maternal fetal medicine, which the website describes as the study of “complicated and high-risk obstetric care, both inpatient and office based.”^54^ Corewell emphasizes the use of models (or simulations) in procedural training for obstetrical emergencies and high-risk medical complications.^55^

Shortly after *Dobbs*, in 2022, the ACGME informed residency programs that “if a program is in a jurisdiction where resident access to this clinical experience is unlawful, the program must provide access to this clinical experience in a different jurisdiction where it is lawful.”^56^ ACGME requirements further stated that “[i]f for some reason a resident is unable to travel to another jurisdiction for clinical experience, the program must provide the resident with a combination of didactic activities, including simulation, and assessment on performing a uterine evacuation and communicating pregnancy options.”^57^ Likewise, the American Board of Obstetrics and Gynecology (ABOG) mandates that graduates fulfill certain requirements in order to take specialty qualifying and certification examinations.^58^ In response to the *Dobbs* decision, ABOG emphasized that “residents seeking ABOG certification [are] required to have satisfactorily completed a minimum of two months, two four-week blocks, or the equivalent of these experiences in family planning … .”^59^

Thus, even in restrictive states, OB/GYN residency programs should incorporate training in abortion. At Morehouse School of Medicine in Georgia (which has a ban on abortion after six weeks),^60^ first-year residents train in high risk obstetric care and family planning.^61^ Before completion of the program, residents complete a rotation in the Emergency Care Center.^62^ However, most programs do not advertise on their websites how they accommodate abortion training per the ACGME’s mandate, and many programs, for which information is not readily accessible, may be out of compliance.^63^

Obstacles to abortion training, to be sure, preceded *Dobbs.* As one study concluded, “[i]nstitutional and programmatic barriers to incorporating training are common and may impose additional restrictions on abortion care provision than dictated by state laws.”^64^ Estimates vary, but pre-*Dobbs* reports from residency directors suggest that only between sixty to sixty-four percent of OB/GYN residency programs offered routine, in-house clinical abortion training.^65^ According to a 2021 study, sixty percent of OB/GYN residents reported routine abortion training, eighteen percent had a clear path to optional training, eleven percent had no clear path to training, and eight percent had no training at all.^66^ Nevertheless, ninety-two percent of OB/GYN residents had access to some level of abortion training in their home programs pre-*Dobbs*, even in states where abortion was restricted.^67^ After *Dobbs*, there has been a significant decrease in residency programs offering routine clinical training in abortion care. Early projections showed that “2,638 of the 6,007 current OB/GYN residents (43.9%) [were] likely or certain to lose access to abortion training in their states.”^68^

ACGME does not require family medicine residency programs to offer abortion training; rather, residents must have clinical hours dedicated to gynecological issues. The American Academy of Family Physicians recommends residents have knowledge of family planning, options counseling, early pregnancy loss and post-abortion symptoms and complications.^69^ As noted by one study, “there has been increasing appreciation of family physician’s [sic] role in abortion provision, especially because family physicians practice in communities where they may be the only health care clinician, and abortion care is well aligned with family medicine’s core values of continuity and whole person care.”^70^ *Dobbs* has impacted medical education for those training in family medicine. In 2023,

although most (63.8%) family medicine residency programs were in states with at least some abortion restrictions, 251 programs (36.2%) were in states with laws protecting abortion. Of the 13,541 residents in accredited US family medicine programs, 3,930 (29%) were training in states that had banned abortion or where abortion was very restricted, and 5,020 residents (37.1%) were in states with protective policies.^71^

Research reveals that residency programs operating in ban states have had decreased student applications and lower levels of participant satisfaction.^72^ The next section briefly reviews those findings before turning to programmatic changes, including resident travel and increased reliance on simulation.

### B. The Consequences of Abortion Bans for Training

Like gaps in training, problems in the availability of obstetric care have long plagued this country. Ban states already had a dearth of practicing OB/GYNs with regional care deserts in the South and Midwest:^73^ “the American College of Obstetricians and Gynecologists (ACOG) estimated that, in 2020, there was a shortage of 8,800 practicing OB/GYNs nationwide, with more than half of all U.S. counties lacking any practicing OB/GYN at all.”^74^ A decrease in applications to OB/GYN residency programs threatens to intensify that shortage.^75^ One study conducted by the Association of American Medical Colleges on the 2022–2023 residency application cycle showed that states with total abortion bans saw the largest decrease in OB/GYN applicants, at 10.5%, or more than double the national average of 5.2%.^76^

*Dobbs*, in addition to shaping where students apply for residency, effects where new OB/GYNs practice. In 2023, of the 349 surveyed residents graduating from abortion training programs, 17.6% indicated that *Dobbs* impacted their practice and fellowship plans.^77^ Indeed, “[r]esidents who before the *Dobbs* decision intended to practice in abortion-restrictive states were eight times more likely to change their practice plans than those who planned to practice in protected states before the *Dobbs* decision.”^78^ And current abortion bans have resulted in physicians moving their practices.^79^ A 2023 survey found that forty percent of OB/GYNs practicing in Idaho, a state with a complete ban,^80^ have left or are considering leaving the state.^81^ Other providers have made the decision to cease providing abortion services and instead offer other types of sexual and reproductive health care.^82^

Studies also demonstrate residents’ deep dissatisfaction with the current system, including moral distress and fears for personal safety.^83^ As one recent report notes, “providers [in ban states] carry the tremendous emotional weight of being forced to limit access to essential patient care, or of risking their licensure or being criminalized. In addition, providers who stay in restrictive states may incur … increased security costs to counter harassment and threats of violence, and the expense of legal consultation or representation to address increased liability.”^84^

To date, attention has focused on residency applications but less has been written about *how* residents in ban-state programs train. In ban states, there is a small patient population seeking in-state abortion services.^85^ Residents rely on treating patients after pregnancy loss,^86^ which typically falls outside of the definition of abortion, or patients with abortions covered by a legal exception,^87^ which often lack clarity as noted in Part I. Said another way, there is an insufficient volume of patients to provide the clinical hours for ban-state training.^88^ Travel for training has been one response to that challenge.

Thus, a core change to medical education has been increased resident travel to programs without restrictive laws, like California or Illinois, where there are more patients. Much of that resident travel has been facilitated by organizations like the Ryan Residency Training Program in Abortion and Family Planning (Ryan Residency Program),^89^ which was established after ACGME first mandated abortion care training for all OB/GYNs. The Ryan Residency Program has helped match residents seeking abortion training with programs across the country.^90^ This process has been and remains complicated: out-of-state rotations take several months to arrange, including completing agreements between programs and meeting in-state as well as institutional requirements for the resident’s placement.^91^ As detailed below, just the paperwork to set up required licensing and insurance coverage is cumbersome, varying based on the home state and the training state.^92^ Residents without the assistance of Ryan Residency Programs often “coordinate clinical opportunities with minimal assistance.”^93^

Just before *Dobbs*, in 2021, eighty-nine Ryan Residency Programs populated sixty percent of residency programs in every region of the United States with one third in states considered hostile to abortion (and three at religiously affiliated hospitals).^94^ After *Dobbs*, the Ryan Residency Program’s work became even more crucial, as now over 120 Ryan Residency Programs help accommodate residents in need of abortion training.^95^

Yet, not everyone can travel for out-of-state training. Residents who cannot travel (as well as those who can) use simulation in order to build skills. Simulation, or using models to teach core procedures, “has been used for many years to augment clinical training in pregnancy termination. Several good models exist, including a papaya-based simulation and didactic scenarios for abortion related complications.”^96^ Programs increasingly rely on simulation in restrictive jurisdictions when clinical experience is not available.^97^ Although simulation and didactics are an important part of training, ACGME has emphasized that simulation cannot “fully replace real-life clinical experience”^98^ — care performed in an office or in an operating room — and “cannot account for the entire context of a patient encounter, simulation is not a substitute for hands-on patient experience.”^99^ Simulation also requires time and resources.^100^

The next Part examines the consequences of post-*Dobbs* residency training for individual learners, for the institutions that host them, and for the future of patient care as well as physician competency. As Amirala S. Pasha and her colleagues wrote, “A gap between the training of clinicians and the needs of the community make care harder to access for all … lead[ing] to the same issues in training and clinical experience that existed prior to *Roe.*”^101^

## IV. The Burdens of the Current System

Of approximately 6000 OB/GYN residents in the United States, 2600 (forty-three percent) are in states with restrictive abortion laws;^102^ over 1200 residents are in states that ban abortion at all stages of gestations.^103^ As noted above, OB/GYNs and training opportunities were scarce in many places before *Dobbs.* ^104^ But more residents, after *Dobbs*, travel or rely on simulation to acquire foundational skills.^105^ People who train to be OB/GYNs now find themselves in an even more fragmented system.

First, graduate education based in part on participant travel is burdensome. For residents in ban states, spending weeks away from their home programs in another state requires time and logistical flexibility. As is true for patients traveling out of ban states for abortion care, the ability to move across state lines depends on various forms of privilege, including resources to cover costs in two places and the ability to pause or delegate obligations at home.^106^ Uprooting one’s life for weeks at a time can cause personal, social, and financial hardships that many residents cannot afford.^107^ In addition, the clinics and hospitals that provide training are predominantly located on the coasts.^108^ The distance between training centers and programs in ban states adds to both the logistical challenges for trainees and tracks “the ever-growing geographic disparities in the distribution of the abortion care workforce.”^109^ Moreover, if residents travel to other programs, they may miss opportunities to hone skills while rotating in their home program and their home programs lose trainees’ labor.^110^ Short-term rotations may not be as effective as learning skills over the course of an entire program.

Second, the post-*Dobbs* stress on institutions that train out-of-state residents is intense. Workforce competition and burnout increasingly characterize the field.^111^ Residents’ demand for clinical hours can exceed the capacity of those programs to provide abortion training for both in-state and out-of-state residents.^112^ As a result, there is increased competition between residents for time with patients.^113^ Add to that logistical and administrative burdens, such as facilitating trainee licenses, that fall to overworked staff members at training institutions. High-volume sites already operate with stretched resources, serving more out-of-state patients and more patients who require complicated care after *Dobbs.* Without additional staff and resources,^114^ cumulative pressures on health centers affect the ability or willingness to train residents.^115^

Third, increased pressures on residency programs could affect the quality of training.^116^ A study by Cara Grimes and her colleagues note the change in how residents now learn counseling skills:

From a nonprocedural perspective, participants identified ways abortion training teaches residents how to counsel patients in challenging situations…Multiple participants described the loss of opportunities to learn effective and compassionate communication skills in difficult encounters, counseling that frequently happens around abortion training.^117^

Fewer educational opportunities could translate into decreased readiness to serve patients needing time-sensitive pregnancy care under restrictive frameworks. In a 2023 study, the research group, Advancing New Standards in Reproductive Health (ANSIRH), found that “post-[*Dobbs*] laws and their interpretations altered the standard of care across these scenarios in ways that contributed to delays, worsened health outcomes, and increased the cost and logistic complexity of care.”^118^ While ANSIRH focused on care withheld due to legal constraints rather than training gaps, delays or denials — whether driven by law, institutional caution, or capacity — can produce adverse patient outcomes,^119^ including increased risk of maternal and infant mortality.^120^

Fourth, evidence suggests that the training some residents receive in ban states is focused on legal risk and overly cautious.^121^ As Lindgren and Oberman have argued, the potential legal consequences of abortion bans, in combination with vague statutory language, have triggered a retreat from the prevailing standard of care when treating pregnant patients.^122^ In the current context, doctors may practice “hesitant” medicine, providing less care than may be optimal because of the threat of civil liability and criminal punishment.^123^ Patient injuries and deaths have been subject of headlines but have not resulted in provider punishment.^124^

What do changes in residency education mean for the standard of care? It may be too early to say with any confidence. But the concern remains that residents trained today may not be equipped to perform essential procedures tomorrow. To help ensure a different trajectory, training across the country must be supported by resources and institutional infrastructure that can address the problems of the present system.

## V. Potential Responses

This article has provided a snapshot of the changes that have emerged in OB/GYN residency programs post-*Dobbs.* The abortion laws that states have enacted in the last few years are unprecedented — total criminal bans, narrow exceptions, the absence of guidance — even as compared to laws that predated *Roe.* ^125^ And what residency programs have done under those laws is an experiment. Residents travel out of state in far greater numbers,^126^ curricula have become piecemeal to a greater extent,^127^ simulation is more prevalent,^128^ and residents and institutions struggle to navigate new systems in the shadow of criminal and civil sanctions.^129^

In this moment of change, responses to the gaps in medical education for OB/GYNs and other health care professionals depend on collaboration, innovation, and flexibility. In that vein, the following recommendations, offered by those working on the ground, seek to address the challenges raised in the previous parts of this article.

### A. Resources for Trainers and Learners

Organizations central to post-*Dobbs* abortion training are translating lessons from their work into actionable best practices. A national summit of abortion providers, professional associations, and non-profit organizations gathered in 2024 to discuss the current landscape of abortion training and, specifically, the “availability, logistical support, legal restrictions, funding, and diversity of the abortion providing workforce.”^130^ The group’s discussion focused on recommendations that could improve post-*Dobbs* resident and practitioner education.^131^

The summit’s experts began by detailing the complications of setting up and coordinating a training program out of state. For example, credentialling standards and insurance requirements differ from place to place; residents’ services billed under supervising physicians can cause difficulties for billing practices.^132^ As demand for training from clinicians and training sites continues to increase, a need has emerged for central databases that share information and training tools. A clearinghouse would be the place to track requests for training; assist institutions in accommodating residents without reinventing the wheel every year and for every program;^133^ and offer training materials, forms, and guidance that could ease the burdens of administration.^134^

Existing partnerships under a ‘hub’ model have developed sample program agreements or letters of affiliation, pre-travel curriculum, modules to accommodate out-of-state learners, and guides for residents. Creating agreements or educational plans and tracking state and professional requirements are time consuming tasks.^135^ The nationwide initiative, the Abortion Training Nexus (ATN), provides these resources to “build a national infrastructure to sustain abortion training through out-of-state partnerships [and] tools.”^136^ ATN seeks to ease the burdens on host sites so that more clinics and hospitals are willing to receive residents.^137^

Funding is vital for groups like ATN, the Ryan Residency Program or the Midwest Access Project (now, Repro TLC), which was founded to coordinate training for family practice residents.^138^ These groups depend on networks of professionals to facilitate training programs, which, as noted, can be expensive for all involved.^139^ Training sites cover costs such as “paying a lawyer to assist with the creation of a training agreement, paying clinical staff overtime to stay later with learners, paying attendings for the time it takes to evaluate learners and provide feedback, hiring additional staff to support the learner experience, and loss of revenue due to longer visits resulting in fewer patients seen per day (lower productivity).”^140^ Additional financial resources could help staff programs that manage the process of training out-of-state residents. The costs related to insurance, equipment, and staff salaries, for example, are often covered by private and state grants.^141^ State funding in places that are supportive of abortion rights, such as in California, New York, Illinois, New Jersey, and Maryland, has supported the expansion of training opportunities.^142^

State-funded programs have prioritized diversifying the pool of providers and better integrating abortion into primary care as well as hospital settings.^143^ In addition to OB/GYNs, as mentioned in Part II, family medicine practitioners can offer obstetric services^144^ and twenty states permit Advance Practice Clinicians (APCs) to provide abortion care.^145^ States like California have invested in APC training at teaching hospitals and local clinics.^146^ For instance, the Reproductive Health Services Corps (RHSC) is a statewide abortion training initiative that focuses on education for a team of professionals — APCs, RNs, MDs, midwives, EMTs, doulas, community health workers, and pharmacists working in a primary care setting.^147^

Expanding educational opportunities also has incorporated training on medication abortion. Medication abortion typically consists of mifepristone followed by misoprostol and is FDA-approved through ten weeks of gestation.^148^ Medication abortion accounts for over half of all abortions in the United States and is increasingly delivered through telehealth.^149^ The growth of virtual clinics followed a 2020 federal district court decision^150^ that temporarily enjoined one of the FDA’s restrictions on mifepristone, which required patients pick up the drug in person.^151^ In December 2021, the FDA lifted that rule,^152^ clearing the way for certified prescribers to furnish mifepristone through mail-order subject to federal and state law.^153^ The average number of abortions is higher now than before *Dobbs*, and mailed medication abortion helps account for that number. The #WeCount study, which has counted abortions since *Roe* was overturned, documents that providers mailed 12,000 packets of medication abortion per month in the last few months of 2024.^154^ Of those, patients in states with total abortion bans or bans at six weeks of gestation received the majority of pills.^155^

Organizations like the Reproductive Health Access Project^156^ and Training in Early Abortion for Comprehensive Healthcare^157^ seek to incorporate medication abortion through telehealth and at Federally Qualified Health Centers or other community-based settings.^158^ The Whole Clinic Training Program at the University of Maryland, Baltimore, “brings expert, hands-on training directly to your site, empowering your entire team, including providers, nurses, administrative staff, and support personnel, with the skills, knowledge, and strategies needed to confidently and effectively integrate safe, high-quality medication abortion services into your practice.”^159^

Having providers teach telehealth counseling, risk assessment, and provision of medication abortion at mobile clinics may increase the opportunities to provide care in states where procedural abortion is limited. In addition, health care professionals will continue to need training on follow-up or post-abortion care as patient travel, care through telehealth, and self-managed abortion increase.^160^ Training programs rely on protocols and guidance to offer various services by a diversity professionals across locations. The next section addresses the role of institutions and professional organizations in assisting those programs.

### B. Institutional Leadership

Institutional actors, such as professional associations, will shape the future of medical education in ban states. Lindgren and Oberman observe, “[t]o care for their patients, clinicians need to know the ‘least-worst best practices;’ [sic] they need the support of their profession to help them distinguish competent from incompetent care.”^161^ ACOG and the American Medical Association came out strongly in support of comprehensive abortion training after *Dobbs.* ^162^ National organizations have since issued guidance on topics like emergency abortion care.^163^ Indeed, the regulation of obstetric emergencies already has come before the Supreme Court.

*Moyle v. United States* ^164^ concerned whether Idaho’s abortion ban, which had no exceptions for medical emergencies, was preempted by the federal Emergency Medical Treatment and Labor Act or EMTALA,^165^ which requires emergency departments to stabilize patients needing emergency care in Medicare-funded hospitals. The Supreme Court ruled that it improvidently granted certiorari and returned the EMTALA issue to lower courts to decide.^166^ At the time of writing, providers are performing EMTALA‑required emergency abortions with protection from criminal sanction in Idaho.^167^ In a separate case, however, the Fifth Circuit held that EMTALA does not authorize the federal government to compel health care providers to perform abortions, even in emergency situations, if abortion is prohibited by state law.^168^

Although national medical organizations agree that abortion can be necessary to save the life or health of the pregnant person,^169^ best practices for addressing both predictable and novel cases could help reduce patient harm. Oberman and her colleagues argue, “[ideally], the Joint Commission on the Accreditation of Hospitals and the National Committee on Quality Assurance (NCQA) would collaborate with professional organizations like ACOG to identify best practices, and adopt them into the relevant accreditation standards and protocols.”^170^ Presently, doctors and their lawyers do not rely on “cross-institutional, multi-state professional networks to set community norms, which would, in turn, set guardrails around defensive medicine” based on consensus-based standards.^171^ Groups with a national remit might be in the best position to gather data and evidence on the clinical care applicable to a range of cases, issuing guidance that could be applied with state laws in mind. Such guidance could inform the content of and the process by which residency education occurs, conferring legitimacy on the decisions of program administrators and participants. Moreover, national leadership might encourage partnerships among doctors and lawyers, who too often act in silos but could work together to train themselves and their colleagues.

Beyond national organizations, state health departments and medical boards also could provide leadership. State departments of health can allocate funding to groups that coordinate logistics for traveling residents, such as the Ryan Residency Program.^172^ Investment in one state’s medical education system would have ripple effects for the entire country: the nature of travel means that states in non-abortion ban medical education systems can help ensure better education for residents who travel to these states and return to home.

State medical boards are the gatekeepers to the profession. One critique of state medical boards has been their historic reticence to wade into controversial topics.^173^ But medical boards could intervene in order to protect their members’ professional interests by helping ensure physicians receive the best education possible. Boards have the power to reaffirm providers’ use of their reasonable medical judgment and doctors’ ethical duty to protect their patients as well as to help dismantle obstacles for out-of-state training through rules and advice.^174^

On the latter, as residents increasingly leave their home states for training, trainee licensing has become a barrier: “[s]tate licensing times vary widely (three–twelve months) and can make cross-state training plans very difficult if not impossible to implement.”^175^ Medical boards (or state entities with similar powers) can facilitate state training licenses for out-of-state learners.^176^ Indeed, “[s]ome states have streamlined visiting-trainee licensure or permit supervised practice under specific training licenses.”^177^ As a longer-term strategy, the Federation of State Medical Boards could advocate for a national system for and a national approach to OB/GYN trainee licensure as it does for licensure portability generally.^178^

Finally, in response to changes in graduate medical education, the ACGME could increase transparency, building on its Resident/Fellow and Faculty Survey, and seek ways to hold non-compliant programs accountable with more publicly-available information.^179^ This might entail developing additional monitoring tools for programs in ban states and asking different kinds of questions about residents’ experiences as well as the skills they have gained.

## VI. Conclusion

Medical education for OB/GYNs has shifted in significant ways. Since *Roe*’s reversal, many states have strict laws that ban abortion. Although many bans contain life or emergency exceptions, they are often narrowly drawn, typically limited to physical conditions, and the health-related language is frequently complex or indeterminate. Doctors and lawyers’ inability to gauge when an exception is met has resulted in delayed and denied care for fear of criminal or civil liability. Under these pressures, perhaps it is not surprising that there has been a drop in applications to OB/GYN residency programs in states with abortion bans.

This article, however, focuses on another concern. Although the availability of abortion training was a problem preceding *Dobbs*, statistics showed most OB/GYN residents had access to some level of training in their home programs. But, since *Dobbs*, the number of residency programs offering routine clinical training in abortion care has decreased. To get the basic education that health care professionals need, many residents in ban states travel for an intensive out-of-state program. While ACGME requires programs to provide access to training, demand is high, multi-week travel is costly and logistically complex, and training-site capacity is limited. This current arrangement cannot serve all users.

Until states no longer ban abortion, the future of abortion training is unclear and what could shift most immediately is the willingness of professionals to work within the present system. OB/GYN residents living in ban states might be increasingly deterred from travel given all the burdens described above. Yet, those residents, who will not or cannot travel, nevertheless are likely to see patients that require abortion care over the course of their careers.

This article has described critical efforts underway to stabilize and to improve graduate medical education for OB/GYN residents post-*Dobbs.* Funding and infrastructure support has helped hospitals and clinics coordinate the burdens of travel. Collaboration among abortion-supportive legislatures, professional organizations, and state boards and health departments has secured resources for cross-state training, both in procedural abortion as well as for medication abortion. But there is more that can be done. Without sustained and increased investment, patients ultimately will pay the price if the number of providers willing and able to offer abortion care continues to decline.

## Footnotes

1 Rachel Crumpler, *Increased Abortion Restrictions Complicate Training, Stoke Worries About Next Generation of OB-GYNs*, N.C. Health News (July 16, 2024) (alteration in original), https://www.northcarolinahealthnews.org/2024/07/16/abortion-restrictions-complicate-training-stoke-worries-about-next-generation-of-obgyns/ [https://perma.cc/B7B7-3F7N] (quoting Jenna Beckham, “an OB-GYN and abortion provider who has been practicing in North Carolina for more than seven years”).

2
*See generally* Dobbs v. Jackson Women’s Health Org., 597 U.S. 215 (2022) (overruling the Court’s decision in *Roe* *v. Wade*).

3
*See generally* Roe v. Wade, 410 U.S. 113 (1973) (establishing a constitutional right to abortion under the Due Process Clause of the Fourteenth Amendment), *overruled by*, Dobbs v. Jackson Women’s Health Org., 597 U.S. 215 (2022).

4
*See, e.g.*, Monique Brouillette, *Abortion Restrictions Could Cause an Ob-Gyn Brain Drain*, Sci. Am. (June 29, 2022), https://www.scientificamerican.com/article/abortion-restrictions-could-cause-an-ob-gyn-brain-drain/ [https://perma.cc/T4HF-ENKM].

5
*See* Melanie Sevcenko, *‘We Weren’t Meant to be Criminals’: the Gynecologists Training Out of State Post*-Roe, The Guardian (June 18, 2023, at 08:00 ET), https://www.theguardian.com/us-news/2023/jun/18/obgyn-doctor-abortion-law-ban [https://perma.cc/VXT2-XTEZ] (“The Ryan Program at Cedars-Sinai in Los Angeles has created a partnership with its Planned Parenthood affiliate, where residents from a state in which abortion is banned can come to undertake their abortion training requirement.”).

6
*See* Arielle Dreher, *OB/GYN Training Programs Try to Adjust to Post*-Dobbs *Reality*, Axios (June 21, 2023), https://www.axios.com/2023/06/21/obgyn-training-programs-try-to-adjust-to-post-dobbs-reality [https://perma.cc/5484-MBL4] (underscoring the increased demand, post-*Dobbs*, for doctors located in ban states to relocate out of state for comprehensive, OB/GYN clinical training).

7
*See* discussion *infra* Section II.B.

8
*See* Mike Connors, *OB/GYN Employment Opportunities in 2025: Market Trends and Essential FAQs*, Barton Assocs. (July 25, 2025), https://www.bartonassociates.com/blog/ob-gyn-employment-opportunities-market-trends-and-essential-faqs/ [https://perma.cc/PA4G-KCFN] (“There’s a growing shortage of OB/GYNs in the United States—projections from the U.S. Health Resources & Services Administration (HRSA) show the country will be short 9,890 OB/GYNs in 2037.”).

9
*See* Anna-Grace Lilly et al., *Our Hands are Tied: Abortion Bans and Hesitant Medicine*, 350 Soc. Sci. & Med., June 2024, at 1, 1.

10
*See* Nadia Sawicki, *Management of Obstetric Emergencies: Evaluating Institutional Policies for Protecting Patients and Physicians in Abortion-Hostile States*, Am. J.L. & Med. (forthcoming 2026).

11 Allison McCann & Amy Schoenfeld Walker, *Tracking Abortion Laws Across the Country*, N.Y. Times (Sep. 8, 2025, at 11:25 ET), https://www.nytimes.com/interactive/2024/us/abortion-laws-roe-v-wade.html [https://perma.cc/P4B4-HDD2] (recognizing that Alabama, Arkansas, Idaho, Indiana, Kentucky, Louisiana, Mississippi, Oklahoma, South Dakota, Tennessee, Texas, and West Virginia ban abortion from conception).

12
*Id.* (highlighting that Florida, Georgia, Iowa, and South Carolina have a six-week gestational limit).

13
*Id.* (explaining that Nebraska and North Carolina ban abortion after twelve weeks).

14
*Id.*

15
*See* Yvonne Lindgren & Michelle Oberman, *Recalibrating Risk Under* Dobbs, 94 Fordham L. Rev. 627, 664 (2025). Before *Dobbs*, Texas enacted a law that creates civil penalties following a successful lawsuit by a private individual. *See, e.g.*, Tex. Health & Safety Code Ann. §§ 171.201–.212 (West 2021).

16 Blake Rocap & Thalia Charles, Life and Health Exceptions Synthesis (Aug. 13, 2025) (on file with the author) (citing Idaho, South Dakota, Texas, Oklahoma, Louisiana, Arkansas); *id.* (recognizing that life exceptions appear to be written two different ways, either as “[p]revent/avert the death” of the pregnant person or “preserve/save the life” of the pregnant person).

17
*Id.* (citing Iowa, Nebraska, North Carolina, South Carolina, West Virginia, and Louisiana’s bans).

18
*Id.*

19
*Id.* (citing language from Indiana, Iowa, and North Carolina’s laws, as well as Idaho’s six-week ban, Mississippi’s total ban, Mississippi’s fifteen-week ban, Texas’ total ban, six-week ban, and its 1925 ban).

20
*Id.*; *see also* Ala. Code § 26-23B-5(a) (2024) (distinguishing that Alabama’s health exception allows abortion when necessary to avert a “serious risk of substantial physical impairment of a major bodily function”).

21 Rocap & Charles, *supra* note 16 (citing Idaho’s six-week ban, Mississippi’s fifteen-week-ban, Texas’ total ban, six-week-ban and 1925 ban, as well as the laws governing Iowa and North Carolina); Tenn. Code Ann. § 39-15-202(f)(1) (2025) (noting that Tennessee permits abortion when necessary to avert or prevent “serious risk of substantial and irreversible physical impairment of a major bodily function of the pregnant woman”).

22 Ind. Code § 16-34-2-0.5 (2024).

23 Miss. Code Ann. § 41-41-37 (2024).

24
*See* Greer Donley & Caroline Kelly, *Abortion Disorientation*, 74 Duke L.J. 1, 66-67 (2024) (arguing that abortion laws and the exceptions under them are confusing, contradictory, and inevitably vague).

25
*See, e.g.*, Kavitha Surana, Lizzie Presser & Andrea Suozzo, *A “Striking” Trend: After Texas Banned Abortion, More Women Nearly Bled to Death During Miscarriage*, ProPublica (July 1, 2025, at 05:00 ET), https://www.propublica.org/article/texas-abortion-ban-miscarriage-blood-transfusions [https://perma.cc/2C4P-W6X7].

26 Maxine Eichner et al., *The Inevitable Vagueness of Medical Exceptions to Abortion Bans*, U.C. Irvine L. Rev. (forthcoming 2025) (manuscript at 1), https://papers.ssrn.com/sol3/papers.cfm?abstract_id=5224731 [https://perma.cc/C8NJ-D574] (“Physicians have contended that the language of medical exceptions are so unclear that they are difficult to apply.”).

27 Nadia Sawicki details the lack of guidance offered by health care institutions in applying medical emergency exceptions. When there has been guidance that is public, it is risk averse and often restrictive. Sawicki, *supra* note 10, pt. II.B.

28 Eleanor Klibanoff, *Texas Adopts Guidance for How Doctors Should Interpret Abortion Ban, The Texas Tribune* (June 21, 2024, at 11:37 CT), https://www.texastribune.org/2024/06/21/texas-medical-board-abortion-guidance-2/ [https://perma.cc/P86X-D27T]; *see* State v. Zurawski, 690 S.W.3d 644, 662–65 (Tex. 2024) (establishing that in Texas the test for determining whether an abortion is legally permissible under the state’s medical emergency exception requires that physicians use their reasonable medical judgment to determine if the patient has a “life-threatening physical condition” and that terminating the pregnancy would avert the risk of death or serious impairment to a major bodily function, which need not depend on risk of imminent harm).

29
*See* S.B. 31, 89^th^ Leg., Reg. Sess. (Tex. 2025).

30 Sawicki, *supra* note 10, at n.15.

31 Lilly et al., *supra* note 9.

32 Lindgren & Oberman, *supra* note 15, at 672.

33 Abortion care is fundamental to managing miscarriage or pregnancy loss — up to one million pregnancies in the United States end in miscarriage each year. Courtney Norris, *Miscarriage is Common. These Researchers are on a Mission to Better Understand Why*, PBS News (June 13, 2024, at 16:14 ET), https://www.pbs.org/newshour/nation/miscarriage-is-common-these-researchers-are-on-a-mission-to-better-understand-why [https://perma.cc/N9F8-LXKL].

34 Katrina Mark et al., *Abortion in Women With Severe Preeclampsia and Eclampsia Prior to 24 Weeks Gestation*, 103 Contraception 420, 420 (2021); Erin Hendricks, Honor MacNaughton & Maricela Castillo MacKenzie, *First Trimester Bleeding: Evaluation and Management*, 99 Am. Fam. Physician 166, 166 (2019); Anna R. Hemnes et al., *Statement on Pregnancy in Pulmonary Hypertension From the Pulmonary Vascular Research Institute*, 5 Pulmonary Circulation 435, 435 (2015).

35
*See, e.g.*, Jericka Duncan, *Family of Georgia Mother Who Died After Delayed Abortion Care Says Amber Thurman Should Still be Alive: “It Was Preventable”*, CBS News (Nov. 1, 2024, at 16:16 ET), https://www.cbsnews.com/news/amber-thurman-delayed-abortion-georgia/ [https://perma.cc/9TWT-WCH4]; Carter Sherman, *Texas Woman Died After Being Denied Miscarriage Due to Abortion Ban, Report Finds*, The Guardian (Oct. 30, 2024, at 13:17 ET), https://www.texastribune.org/2024/11/27/texas-abortion-death-porsha-ngumezi/ [https://perma.cc/3NU8-WY7C]; Pooja Salhotra, *Texas OB-GYNs Urge Lawmakers to Change Abortion Laws After Reports on Pregnant Women’s Deaths*, The Texas Tribune (Nov. 3, 2024, at 16:00 CT), https://www.texastribune.org/2024/11/03/texas-ob-gyn-letter-abortion-laws/ [https://perma.cc/K7WW-45FG].

36 Jody E. Steinauer et al., *Abortion Training in US Obstetrics and Gynecology Residency Programs*, 86 Am. J. Obstetrics & Gynecology e1 (2018) (citing ACGME Program Requirements for Graduate Medical Education in Obstetrics and Gynecology and American College of Obstetricians and Gynecologists Committee Opinions).

37
*See generally* Lori Freedman, Willing and Unable: Doctors’ Constraints in Abortion Care (2010) (examining physicians’ experiences navigating institutional, legal and cultural limits on providing abortions).

38 Julia Strasser et al., *Workforce Providing Abortion Care and Management of Pregnancy Loss in the US*, 182 JAMA Internal Med. 558, 558 (2022).

39 Nicole Dube, Conn. Off. of Leg. Rsch., 2022-R-0167, States Allowing Non-Physicians to Provide Abortion Services 1–2 (2022).

40 “[I]n 1996, the ACGME added participation in abortion care to core requirements for the OB/GYN residency, stating ‘programs must provide training or access to training in the provision of abortions, and this must be a part of the planned curriculum.’” Anitra D. Beasley et al., *New Gaps and Urgent Needs in Graduate Medical Education and Training in Abortion*, 98 Academic Med. 436, 437 (2023).

41 Strasser et al., *supra* note 38, at 559.

42 ACGME, Program Requirements for Graduate Medical Education in Obstetrics and Gynecology 26–27 (2025), https://www.acgme.org/globalassets/pfassets/programrequirements/2025-reformatted-requirements/220_obstetricsgynecology_2025_reformatted.pdf [https://perma.cc/B2T3-TQYY].

43 The Nat’l Acads. of Scis., Eng’g, & Med., The Safety and Quality of Abortion Care in the United States 106 (2018) [hereinafter Safety and Quality].

44
*ACGME Frequently Asked Questions (FAQs)*, Accreditation Council for Graduate Med. Educ., https://www.acgme.org/about/acgme-frequently-asked-questions/ [https://perma.cc/JC7S-KK6Y] (last visited Sep. 24, 2025).

45 42 U.S.C. § 238n (the Coats-Snowe Amendment); Kristina Tocce & Britt Severson, *Funding for Abortion Training in Ob/Gyn Residency*, 14 Am. Med. Ass’n J. Ethics 113, 113-14 (2012) (describing the Coats-Snowe Amendment). Otherwise, teaching institutions without accreditation risk severe financial consequences, such as losing eligibility for Medicare funding for graduate medical education. 42 U.S.C. § 1395ww(h), (d)(5)(B) (establishing Direct and Indirect Medical Education payments); 42 U.S.C. § 413.75(b (defining “approved medical residency program” as one approved by the ACGME).

46 Beasley et al., *supra* note 40, at 437. A two-year fellowship in family planning, established in 1991, helps learners build their skills in complex abortion and contraception. The fellowship has been offered at more than 30 sites. In 2018, the American Board of Medical Specialties and American Board of Obstetrics and Gynecology approved subspecialty certification for complex family planning. *Id.*

47
*Stanford Obstetrics and Gynecology Rotation Schedule*, Stanford Med., https://med.stanford.edu/obgyn/education/residency/rotations.html [https://perma.cc/9MZB-3HGR] (last visited Oct. 9, 2025).

48 Safety and Quality, *supra* note 43, at 108; *Stanford Obstetrics and Gynecology Rotation Schedule*, *supra* note 47.

49
*Stanford Obstetrics and Gynecology Rotation Schedule*, *supra* note 47.

50
*Swedish OB/GYN Residency Rotation Schedule*, Swedish Ob/Gyn Residency, https://swedishobgynresidency.com/rotation-schedule/ [https://perma.cc/YAQ5-E55N] (last visited Oct. 9, 2025).

51
*Id.*

52
*Obstetrics & Gynecology Residency Clinical, Research & Didactic Training*, NYU Langone Health, https://med.nyu.edu/departments-institutes/obstetrics-gynecology/education/residency/clinical-research-didactic-training [https://perma.cc/C4JB-C8QE] (last visited Sep. 26, 2025).

53
*Id.*

54
*Obstetrics and Gynecology Residency*, Corewell Health, https://corewellhealth.org/graduate-medical-education/west-michigan/residencies/obstetrics-and-gynecology [https://perma.cc/A6ZG-KC7J] (last visited Sep. 26, 2025).

55
*Emergency Medicine: Simulation*, Corewell Health, https://corewellhealth.org/graduate-medical-education/southeast-michigan/residencies/royal-oak/emergency-medicine/simulation [https://perma.cc/8SUH-TWRA] (last visited Oct. 9, 2025).

56 ACGME, *supra* note 42, at 27.

57
*See* Physicians for Reprod. Health, Abortion Training: Policy Primer 3 (2024), https://prh.org/wp-content/uploads/2024/10/policy-fact-sheet-abortion-training.pdf#:~:text=Page%203,assessment%20on%20performing%20a%20uterine [https://perma.cc/5DXJ-VYDX] (quoting the 2022 ACGME guidance). The September 2025 guidance issued by ACGME no longer contains this sentence. *See* ACGME, *supra* note 42.

58
*See OB-GYN Standards for Certification*, Am. Bd. of Obstet. & Gynecol. (Sep. 21, 2022), https://www.abog.org/get-certified/specialty-certification/overview/ob-gyn-standards-for-certification [https://perma.cc/9A4D-ANY2].

59
*Id.* (alteration in original).

60
*See* McCann & Walker, *supra* note 11; *supra* text accompanying notes 11–13.

61
*See OB/GYN Curriculum*, Morehouse Sch. of Med., https://www.msm.edu/Education/GME/OBGYNResidencyProgram/OBGYNCurriculum.php [https://perma.cc/P5M3-YSFN] (last visited Sep. 22, 2025).

62
*See OBGYN Residency Program: Rotation Schedule*, Morehouse Sch. of Med., https://www.msm.edu/Education/GME/OBGYNResidencyProgram/RotationSchedule.php [https://perma.cc/JN4S-RMGV] (last visited Sep. 22, 2025).

63 “Only two-thirds of obstetrics and gynecology residency programs publicize abortion training information. Almost half of all programs in states with abortion bans do not share this information, revealing a need for improved transparency to better inform residency applicant decision-making.” Aishwarya Karlapudi, Radhika Rible & Kavita Vinekar, *Transparency Around Abortion Training on Obstetrics and Gynecology Residency Program Websites*, 130 Contraception, Feb. 2024, at 1, 1.

64 Beasley et al., *supra* note 40, at 437 (alteration in original).

65 “As of 2014, only 64% of residency program directors reported dedicated abortion training within scheduled rotations, with 31% reporting optional or unscheduled abortion care training and 5% reporting no abortion training.” *Id.*; *see generally* Freedman, *supra* note 37 (detailing the gaps in medical education for physicians who want to provide abortions but are unable to do so for educational, financial, and logistical reasons).

66 Sarah Horvath et al., *Resident Abortion Care Training and Satisfaction: Results from the 2020 Council on Resident Education in Obstetrics and Gynecology In-Training Examination Survey*, 138 Obstet. Gynecol. 472, 472–73 (2021).

67 Amirala S. Pasha, Daniel Breitkopf & Gretchen Glaser, *The Impact of* Dobbs *on US Graduate Medical Education*, 51 J.L., Med. & Ethics 497, 500 (2022).

68
*Id.* (alteration in original).

69
*See* Sarah Wulf et al., *Implications of Overturning* Roe v Wade *on Abortion Training in US Family Medicine Residency Programs*, 21 Ann. Fam. Med. 545, 545 (2023); ACGME, ACGME Program Requirements for Graduate Medical Education in Family Medicine 33 (2025), https://www.acgme.org/globalassets/pfassets/programrequirements/2025-reformatted-requirements/120_familymedicine_2025_reformatted.pdf [https://perma.cc/3PP6-65DU] (requirement for learning “family planning, contraception, and options education for unintended pregnancy”); Am. Acad. of Fam. Physicians, Recommended Curriculum Guidelines for Family Medicine Residents, Women’s Health and Gynecologic Care 4–6 (2023), https://www.aafp.org/dam/AAFP/documents/medical_education_residency/program_directors/womens-health-and-gynecologic-care.pdf [https://perma.cc/3D3N-N6GX].

70 Wulf et al., *supra* note 69, at 545.

71
*Id.* at 546.

72
*See* Kendal Orgera, Hasan Mahmood & Atul Grover, *Training Location Preferences of U.S. Medical School Graduates Post* Dobbs v. Jackson Women’s Health, AAMC (Apr. 23, 2023), https://www.aamc.org/about-us/mission-areas/health-care/training-location-preferences-us-medical-school-graduates-post-dobbs-v-jackson-women-s-health [https://perma.cc/MZC7-D944]; Angela Du, Rachel M. Smith & Katherine Rivlin, *Satisfaction with Abortion Training in an Abortion-Restrictive State*, 15 J. Grad. Med. Educ. 551, 551 (2023).

73
*See* U.S. Health Res. & Servs. Admin., Projections of Supply and Demand for Women’s Health Service Providers: 2018-2030, at 7 (2021), https://bhw.hrsa.gov/sites/default/files/bureau-health-workforce/data-research/projections-supply-demand-2018-2030.pdf [https://perma.cc/K9MC-DC6R].

74 Nat’l P’ship for Women & Fams. & Physicians for Reprod. Health, Dobbs *’* Erosion of the Health Care Workforce: Harm to Providers and Patients 2 (2024) [hereinafter Nat’l P’ship for Women & Fams], https://nationalpartnership.org/wp-content/uploads/dobbs-erosion-health-care-workforce.pdf [https://perma.cc/SGZ6-83Z5]; Linda Marsa, *Labor Pains: The OB-GYN Shortage*, AAMC (Nov. 15, 2018), https://www.aamc.org/news/labor-pains-ob-gyn-shortage [https://perma.cc/3427-RZCP].

75
*See id.* at 10. To be clear, the OB/GYN residency match process remains highly competitive, with more applications than positions available. *See* Nat’l Resident Matching Program, Results and Data: 2025 Main Residency Match 2 (2025), https://www.nrmp.org/match-data/2025/05/results-and-data-2025-main-residency-match/ [https://perma.cc/3P7L-NMKM]. A matching process requires students to match with a program based in part on rank order lists. The applicants have an opportunity to influence the process at two stages — one during interviews and two when drafting their own rank order list. *See Ranking Programs in the Main Residency Match*, Nat’l Resident Matching Program, https://www.nrmp.org/residency-applicants/rank-your-programs-main [https://perma.cc/R7UK-AXFP] (last visited Sep. 27, 2025). However, once matched to a program, applicants commit to attend it. *See id.*

76
*See* Orgera et al., *supra* note 72.

77 Alexandra L. Woodcock et al., *Effects of the* Dobbs v. Jackson Women’s Health Organization *Decision on Obstetrics and Gynecology Graduating Residents’ Practice Plans*, 142 Obstet. Gynecol. 1105, 1105 (2023).

78
*Id.* (alteration in original).

79
*See* Nat’l P’ship for Women & Fams, *supra* note 74, at 4.

80
*See supra* note 11 and accompanying text.

81 Sarah Varney, *Idaho’s Strict Abortion Laws Create Uncertainty for OB-GYNs in the State*, PBS News (May 1, 2023, at 18:30 ET), https://www.pbs.org/newshour/show/idahos-strict-abortion-laws-create-uncertainty-for-ob-gyns-in-the-state [https://perma.cc/T463-DPST].

82
*See* Nat’l P’ship for Women & Fams, *supra* note 74, at 4.

83
*See* Stephanie J. Lambert, Sarah K. Horvath & Rachel S. Casas, *Impact of the *Dobbs* Decision on Medical Education and Training in Abortion Care*, 33 Women’s Health Issues 337, 338 (2023); *see also* Du, Smith & Rivlin, *supra* note 72, at 556.

84 Nat’l P’ship for Women & Fams, *supra* note 74, at 6 (alteration in original).

85
*See* Kari White et al., *Association of Texas’ 2021 Ban on Abortion in Early Pregnancy With the Number of Facility-Based Abortions in Texas and Surrounding States*, 328 [J]AMA 2048, 2048 (2022) (finding significant decrease in number of abortions obtained by Texas residents both in and out of Texas following passage of SB8 in 2021).

86
*See* Strasser et al., *supra* note 38.

87
*See id.*

88
*See* Pasha, Breitkopf & Glaser, *supra* note 67, at 500 (“After *Dobbs*, low volumes of abortion procedures may lead to the same issues in training and clinical experience that existed prior to *Roe.*”).

89
*About the Ryan Program*, Kenneth J. Ryan Training Program in Abortion & Fam. Plan., https://ryanprogram.org/home/overview [https://perma.cc/7PD3-2SWW] (last visited Sep. 19, 2025). The Ryan Residency Program has twenty-one partnerships now operating to train residents and, in the course of its history, the Program has trained over 15,000 residents. Residents in southeastern states with abortion bans travel, for example, to Illinois hospitals and clinics. The majority of Texas residents travel to California programs. *Id.*

90
*Id.*

91
*See* Jema K. Turk et al., *Out-of-State Abortion Training Rotation Residents in States with Limited Access*, 1 Obstetrics & Gynecology Open 1, 2 (2024) (“The logistics of establishing these rotations were complex. The process of matching programs took an average of 5 months before the first resident could travel. The processes included negotiating contracts and agreements between institutions, coordinating rotation schedules with host institutions, supporting individual learners’ applications, and making travel plans, among other details … .”).

92
*Id.* at 3.

93 Beasley et al., *supra* note 40, at 438.

94
*Id.* at 437.

95
*See* Turk et al., *supra* note 91, at 1.

96 Pasha, Breitkopf & Glaser, *supra* note 67, at 501.

97 Beasley et al., *supra* note 40, at 438.

98 Pasha, Breitkopf & Glaser, *supra* note 67, at 501.

99
*Id.*; Beasley et al., *supra* note 40, at 438.

100 Beasley et al., *supra* note 40, at 438.

101 Pasha, Breitkopf & Glaser, *supra* note 67, at 500.

102 Cara L. Grimes et al., *Anticipated Impact of *Dobbs v Jackson Women’s Health Organization* on Training of Residents in Obstetrics and Gynecology: A Qualitative Analysis*, 15 J. Grad. Med. Educ. 339, 339 (2023).

103
*Ryan Program Locations*, Kenneth J. Ryan Training Program in Abortion & Fam. Plan., https://ryanprogram.org/home/overview/ryan-program-locations/ [https://perma.cc/4BAQ-HDW2] (last visited Nov. 21, 2025).

104
*See* Beasley et al., *supra* note 40, at 437.

105
*Abortion Training and Education in a Post-*Dobbs* Landscape*, Am. Coll. of Obstetricians & Gynecologists (Feb. 6, 2025), https://www.acog.org/clinical-information/policy-and-position-statements/position-statements/2025/abortion-training-and-education-in-a-post-dobbs-landscape [https://perma.cc/K33A-4CDR].

106
*See* Ortal Wasser, Lauren J. Ralph, Shelby Kaller & Antonia Biggs, *Catastrophic Health Expenditures for In-State and Out-of-State Abortion Care*, JAMA Network Open, Nov. 8, 2024, at 1, 7 (describing abortion-related expenses for residents of states with and without abortion bans).

107
*See* Pasha, Breitkopf & Glaser, *supra* note 67, at 499.

108 Allison Whitney, Strategies for Creating a Wider Variety of Abortion Providers in a Post *Dobbs* Environment: Recommendations from a National Summit 5 (Apr. 27, 2024) (unpublished manuscript) (on file with the author) (noting exceptions like Chicago and Kansas City); *About*, Abortion Training Nexus, https://www.abortiontrainingnexus.org/about [https://perma.cc/J6CV-H9EC] (last visited Nov. 21, 2025) (depicting where residents travel, including Illinois).

109 Whitney, *supra* note 108, at 5.

110 Pasha, Breitkopf & Glaser, *supra* note 67, at 500.

111 Mohammad Najib et al., *Brain-Drain Phenomenon Among Healthcare Workers*, Int’l J. Pub. Health & Clinical Scis., May 2019, at 90, 92, 96.

112
*See* Grimes et al., *supra* note 102, at 344.

113
*See* Turk et al., *supra* note 91, at 2.

114
*See id.*

115 Amir Mian et al., *Medical Student and Resident Burnout: A Review of Causes, Effects, and Prevention*, J. Fam. Med. & Disease Prevention, 2018, at 1, 2.

116 Grimes et al., *supra* note 102, tbl. 2, 342–43 (highlighting concerns in OB/GYN education post-*Dobbs* in eight focus areas: competency, provision of reproductive health care, residency selection, inequity in training, alternative training, law-based rather than evidence-based medicine, morality and ethics, and uncertainty and future steps).

117
*Id.* at 341.

118
*See* Daniel Grossman et al., Care Post-Roe: Documenting Cases of Poor Quality Care Since the *Dobbs* Decision, Advancing New Standards in Reproductive Health (2023) (alteration in original), https://www.ansirh.org/sites/default/files/2023-05/Care%20Post-Roe%20Preliminary%20Findings.pdf [https://perma.cc/9XNG-2D93].

119
*See id.* at 4.

120 Jacqueline Howard, *Maternal and Infant Death Rates are Higher in States that Ban or Restrict Abortion, Report Says*, CNN Health (Dec. 16, 2022, at 08:29 ET), https://www.cnn.com/2022/12/14/health/maternal-infant-death-abortion-access/index.html [https://perma.cc/7N6V-WRME] (detailing already high maternal and infant mortality rates have increased twofold in states that ban abortion post-*Dobbs*).

121
*See* Olivia Goldhill, *After *Dobbs*, U.S. Medical Students Head Abroad for Abortion Training* *No Longer Provided by their Schools*, Statnews (Oct. 18, 2022), https://www.statnews.com/2022/10/18/medical-students-heading-abroad-for-abortion-training/ [https://perma.cc/8E87-KMRA].

122 Lindgren & Oberman, *supra* note 15, at 666.

123 Lilly et al., *supra* note 9, at 2–7.

124
*See* Selena Simmons-Duffin, *Doctors Who Want to Defy Abortion Laws Say It’s Too Risky*, NPR (Nov. 23, 2022, at 05:01 ET), https://www.npr.org/sections/health-shots/2022/11/23/1137756183/doctors-who-want-to-defy-abortion-laws-say-its-too-risky [https://perma.cc/VF3F-9QRW]; *see also* Kavitha Surana et al., *Are Abortion Bans Across America Causing Deaths?*, ProPublica (Dec. 18, 2024, at 05:00 ET), https://www.propublica.org/article/abortion-bans-deaths-state-maternal-mortality-committees [https://perma.cc/4LQQ-6ATK].

125
*See* Reva Siegel & Mary Ziegler, *Abortion’s New Criminalization: A History-and-Tradition Right to Health Care Access after* Dobbs, 111 Va. L. Rev. 413, 416 (2025) (“far from returning to the past, the criminalization regime emerging after *Dobbs* is in critical ways far more punitive”).

126
*Abortion Training and Education in a Post-*Dobbs* Landscape*, *supra* note 105.

127
*Id.*

128
*See* Rachel Rebouché, *How *Dobbs* Created an Abortion Training* Crisis, The Nation (Oct. 14, 2024), https://www.thenation.com/article/society/how-dobbs-created-an-abortion-training-crisis [https://perma.cc/KG3U-MFY4].

129
*See* Julian Polaris, Naomi Newman, & Allie Levy Chafetz, *Post-*Dobbs* Considerations for Provider Organizations Navigating State Restrictions on Abortion*, Manatt (Aug. 28, 2022), https://www.manatt.com/insights/white-papers/2022/post-dobbs-considerations-for-provider-organizatio [https://perma.cc/42YF-7MAQ].

130 Whitney, *supra* note at 108, at 3.

131
*See id.*

132
*See* Turk et al., *supra* note 91.

133 “Currently there are a number of organizations and projects providing logistical support and funding to help to fill this gap on a national level; Midwest Access Project (MAP), Nurses for Sexual and Reproductive Health (NSRH), TEACH as part of the Reproductive Health Service Corps (RHSC) and the Clinical Abortion Training Centers project (CATC). These projects work with individual abortion clinics (both Planned Parenthood affiliates and independent clinics) by accepting learner applications, vetting learners, and providing logistical and financial support to learners prior to learners arriving on site.” *Id.* Whitney, *supra* note at 108, at 9.

134 “There are already a number of excellent resources to support the development of an effective training program including those from organizations like TEACH, which provides a guide for training in first trimester abortion, and My Tip Report, which is an evaluation tool that has been configured specifically for abortion skills evaluation. Planned Parenthood also has an Abortion Training Toolkit available that could be revised to meet the needs of independent clinics as well … .” *Id.* at 10.

135
*See Understanding the Credentialing Process*, CompHealth (Jan. 12, 2022), https://comphealth.com/resources/understanding-the-credentialing-process [https://perma.cc/7JW6-8PRU].

136
*Partnership*, Abortion Training Nexus, https://www.abortiontrainingnexus.org/partnership [https://perma.cc/EJ49-MVS9] (last visited Nov. 21, 2025) (offering samples of Program Letter of Agreement, for example).

137
*About*, *supra* note 108. Few hospitals provide in-house training, but most work with clinics, such as Planned Parenthood affiliates. *See Family Planning Division*, Brigham & Women’s Hosp, https://www.brighamandwomens.org/obgyn/family-planning/family-planning-division [https://perma.cc/NP4R-WHFJ] (last visited Nov. 11, 2025).

138
*See* Jody Steinauer et al., *The Benefits of Family Planning Training: A 10-Year Review of the Ryan Residency Training Program*, 88 Contraception 275, 277 (2013). The partners in training for MAP are high volume abortion clinics with a mix of Planned Parenthood affiliates and independent clinics; most trainees are looking for training opportunities in procedural competency as medication abortion becomes more prevalent. *See Protect Access to Clinical Training in Abortion Care*, Midwest Access Project (June 30, 2022), https://wetheaction.org/projects/1670-protect-access-to-clinical-training-in-abortion-care [https://perma.cc/WP3L-TC8U].

139
*Protect Access to Clinical Training in Abortion Care*, *supra* note 138.

140 Whitney, *supra* note at 108, at 9. For the trainee, as detailed in above, costs can include travel, housing, food, and license fees.

141
*A Practical Guide to Understanding Restrictions on Use of Federal Funds for Abortion Services*, Reprod. Health Access Project, www.reproductiveaccess.org/wp-content/uploads/2023/10/Practical-Guide-to-Understanding-Restrictions-on-Federal-Funds-for-Abortion-Providers.pdf [https://perma.cc/55UJ-D6AR] (last visited Oct. 22, 2025).

142
*What California is Doing to Protect Abortion Access*, Cal. Dep’t of Pub. Health, https://www.cdph.ca.gov/Programs/OHE/abortion/Pages/your-rights/state-action.aspx [https://perma.cc/MCH3-HMYQ] (last visited Sep. 28, 2025); *Single Source Procurement: New York State Abortion Access Program (NYSAAP)*, N.Y. State Dep’t of Health (Nov. 2023), https://www.health.ny.gov/funding/single_source/nysaap.htm [https://perma.cc/XK73-2VV8]; Press Release, Ill. Governor’s Off., Gov. Pritzker Signs Sweeping Reproductive Rights Protections Into Law, Ill. Governor’s Off. (Jan. 13, 2023), https://www.illinois.gov/news/release.html?releaseid=25906 [https://perma.cc/6KDZ-5Q5S?type=image]; *Governor Murphy Signs Legislation to Protect Reproductive Health Care*, Governor’s Off. of N.J. (July 1, 2022), https://www.nj.gov/governor/news/news/562022/20220701a.shtml [https://perma.cc/L3RY-7STP]; *Governor Moore Signs Proclamation to Enshrine Reproductive Freedom in Maryland’s Constitution*, Off. of Governor Wes Moore (Jan. 17, 2025), https://governor.maryland.gov/news/press/pages/governor-moore-signs-proclamation-to-enshrine-reproductive-freedom-in-maryland%E2%80%99s-constitution.aspx. [https://perma.cc/G5JZ-C3WF].

143 Kimya Forouzan & Isabel Guarnieri, *State Policy Trends 2023: In the First Full Year Since *Roe* Fell, a Tumultuous Year for Abortion and Other Reproductive Health Care*, Guttmacher Inst. (Dec. 19, 2023), https://www.guttmacher.org/2023/12/state-policy-trends-2023-first-full-year-roe-fell-tumultuous-year-abortion-and-other [https://perma.cc/784X-SCSF].

144
*See Reproductive Health & Advocacy Fellowship*, Univ. of Wash. Dep't. Fam. Med., https://familymedicine.uw.edu/residency/fellowships/reproductive-health-advocacy-fellowship/ [https://perma.cc/6LYH-FCSD] (last visited Sep. 28, 2025) (focusing on developing clinical, advocacy, and leadership training in reproductive health among family medicine residents).

145 Currently, APCs are legally providing procedural and medication abortion care in twenty states. Dube, *supra* note 39, at 2. APCs can provide medication abortion in three states. Whitney, *supra* note 108, at 3.

146
*Public Policy*, Training in Early Abortion for Comprehensive Healthcare, https://teachtraining.org/publicpolicy#ca [https://perma.cc/YN2T-TX63] (last visited Sep. 28, 2025); Whitney, *supra* note at 108, at 3.

147
*About RHSC*, Reprod. Health Servs. Corps, https://reprohealthservicecorps.org/about [https://perma.cc/3LR8-YH2Q] (last visited Sep. 28, 2025).

148
*See generally* David S. Cohen et al., *Abortion Pills*, 76 Stan. L. Rev. 317, 320–24 (2024) (describing the regulation and uptake of mailed medication abortion).

149 Talia Curhan, *Medication Abortion*, Guttmacher Inst. (Apr. 23, 2025), https://www.guttmacher.org/state-policy/explore/medication-abortion [https://perma.cc/UV7D-K7YP].

150
*See* Am. Coll. of Obstetricians & Gynecologists v. FDA, 472 F. Supp. 3d 183, 215–16 (D. Md. 2020) (striking down in-person requirements for medication abortion as a “substantial obstacle” for patients during the COVID-19 pandemic).

151
*The FDA’s Decision Lifting the Burdensome Restriction on Mifepristone during the Pandemic: What You Need to Know*, Am. Coll. of Obstetricians & Gynecologists (Apr. 21, 2021), https://www.acog.org/news/news-articles/2020/07/courts-order-lifting-burdensome-fda-restriction [https://perma.cc/TM69-MW44].

152 U.S. Food & Drug Admin., Information about Mifepristone for Medical Termination of Pregnancy Through Ten Weeks Gestation 2-3 (2021).

153
*See* Cohen et al., *supra* note 148, at 330–31 (describing how the removal of the in-person restriction changed the uptake of medication abortions by expanding access to abortion by mail).

154
*#WeCount Report, April 2022 to December 2024*, Soc’y of Fam. Plan. (June 23, 2025), https://societyfp.org/research/wecount/wecount-december-2024-data/ [https://perma.cc/63JN-LWLE].

155 Abigail R.A. Aiken et al., *Provision of Abortion Medications Using Online Asynchronous Telemedicine Under Shield Laws in the US*, 334 [J]AMA 1388, 1388–89 (Oct. 21, 2025).

156
*Integrating Medication Abortion into Primary Care*, Reprod. Health Access Project, https://www.reproductiveaccess.org/abortion/pc-abortion-resources/ [https://perma.cc/5MKY-E3TD] (last visited Sep. 28, 2025).

157
*Abortion Training Workshops and Simulations*, Training in Early Abortion for Comprehensive Healthcare, https://teachtraining.org/workshops [https://perma.cc/32PY-YQBX] (last visited Sep. 28, 2025).

158
*See Integrating Medication Abortion into Primary Care*, *supra* note 156; *see also Integrate Reproductive Health Initiative*, Training in Early Abortion for Comprehensive Healthcare, https://teachtraining.org/integrate [https://perma.cc/3423-QBA3] (last visited Sep. 28, 2025).

159
*Whole Clinic Training Program*, Univ. of Md. Balt., https://www.umaryland.edu/lifelong-learning/reproductive/training/whole-clinic-training-program/ [https://perma.cc/S5UZ-LGNQ] (last visited Nov. 21, 2025).

160 Lisa H. Harris & Daniel Grossman, *Complications of Unsafe and Self-Managed Abortion*, 382 New Engl. J. Med. 1029, 1033–38 (2020). This article does not address the important question of how follow-up training happens.

161 Lindgren & Oberman, *supra* note 15, at 677.

162
*Abortion Training and Education in a Post-*Dobbs* Landscape*, *supra* note 105; Kevin B. O’Reilly, *AMA Holds Fast to Principle: Reproductive Care is Health Care*, Am. Med. Ass’n (Nov. 17, 2022), https://www.ama-assn.org/public-health/population-health/ama-holds-fast-principle-reproductive-care-health-care. [https://perma.cc/JRG6-FGF5].

163
*Abortion Training and Education in a Post-*Dobbs* Landscape*, *supra* note 105.

164 Moyle v. United States, 603 U.S. 324 (2024).

165 Emergency Medical Treatment and Active Labor Act, 42 U.S.C. § 1395dd(a).

166 Moyle v. United States, 603 U.S. 324, 325 (2024) (Jackson, J., dissenting) (“Will this court just have a do-over, rehearing and rehashing the same arguments we are considering now, just at a comparatively more convenient point in time?”). *Id.* at 344.

167
*Medical Emergency Exceptions to Idaho’s Abortion Ban*, Ctr. for Reprod. Rts. (Oct. 29, 2025), https://reproductiverights.org/cases/adkins-v-state-of-idaho/#:~:text=On%20April%2011%2C%20an%20Idaho,prosecution%20for%20providing%20that%20care [https://perma.cc/44M7-WRAL]; *Moyle v US; Idaho v US*, Milken Inst. of Pub. Health (Oct. 22, 2024), https://hpmmatters.publichealth.gwu.edu/moyle-v-us-idaho-v-us [https://perma.cc/5A9X-NEEA].

168 Texas v. Becerra, 89 F.4th 529, 535 (5th Cir. 2024).

169 Stella M. Dantas, *ACOG Urges Protections for Emergency Abortion Care*, Am. Coll. of Obstetricians & Gynecologists (June 27, 2024), https://www.acog.org/news/news-releases/2024/06/acog-urges-protections-for-emergency-abortion-care [https://perma.cc/QG7J-DWNB].

170 Michelle Oberman et al., *Evidence Based Medicine After* Dobbs, New Eng. J. Med. (forthcoming 2025) (manuscript at 1, 5) (on file with the author) (alteration in original).

171
*Id.* (manuscript at 3).

172 For instance, Illinois’s health department was instrumental in a state award of financial support for MAP. *See IDPH Awards $2 Million in Training Grants to 3 Groups to Improve Reproductive Health Services*, Ill. Dep’t of Health (Apr. 11, 2024), https://dph.illinois.gov/resource-center/news/2024/april/2024-04-11---idph-awards--2-million-in-training-grants-to-3-grou.html [https://perma.cc/H7V4-3S36].

173 Dov Fox, *Medical Disobedience*, 136 Harv. L. Rev. 1030, 1049 (2023) (explaining the shortcomings of state medical boards in abortion law and policy).

174 The 2021 revised ethical principles of the AMA state: “A physician shall respect the law and also recognize a responsibility to seek changes in those requirements which are contrary to the best interests of the patient.” *AMA Principles of Medical Ethics*, Am. Med. Ass’n (June 2021), https://code-medical-ethics.ama-assn.org/principles [https://perma.cc/5V2D-WCNG]. *See also* Abigail Weinberg, *Doctors Should Break the Law to Offer Abortions Sometimes, Says New Guidance From Medical Leaders*, Mother Jones (Nov. 20, 2022), https://www.motherjones.com/politics/2022/11/abortion-rights-american-medical-association-guidance-doctors-ethics/ [https://perma.cc/B5WV-MNG4] (“In new guidance, the American Medical Association tried to clear things up, advising that medical providers should act in the interest of their patients’ well-being—even if it means violating the law.”).

175 Austin Fast, *Nurses are Waiting Months for Licenses as Hospital Staffing Shortages Spread*, Nat’l Pub. Radio, (Mar. 10, 2022, at 05:08 ET) (alteration in original), https://www.wqcs.org/2022-03-10/nurses-are-waiting-months-for-licenses-as-hospital-staffing-shortages-spread [https://perma.cc/TCS9-SD9N].

176 Drew Carlson & James N. Thompson, *Policy Forum: The Role of State Medical Boards*, Am. Med. Ass’n J. Ethics, Apr. 2005, at 1, 4.

177
*See Postgraduate Training Licensees*, Med. Bd. of Cal. (Sep. 9, 2025), https://www.mbc.ca.gov/Licensing/Postgraduate-Training-Licensees/Apply.aspx [https://perma.cc/D2VK-6HPD]; Ill. Dep’t of Fin. & Pro. Regul., Instruction Sheet: Physician and Surgeon, Temporary Licensure, Limited Temporary Licensure, Transfer of Temporary Licensure, Extension of Temporary Licensure 2, 11 (2025) (setting out the procedure for acquiring a resident training license).

178
*See U.S. Medical Regulatory Trends and Actions*, FSMB, https://www.fsmb.org/u.s.-medical-regulatory-trends-and-actions/ [https://perma.cc/UN47-4ZFX] (last visited Nov. 23, 2025) (discussing licensure portability, the Interstate Medical Licensure Compact (for fully licensed physicians), and harmonization of trainee-licensure processes).

179
*See Resident/Fellow and Faculty Survey*, ACGME, https://www.acgme.org/data-systems-technical-support/resident-fellow-and-faculty-surveys/ [https://perma.cc/Z8Y2-EEX4] (last visited Nov. 23, 2025) (“Resident/Fellow and Faculty Surveys are used to monitor graduate medical clinical education and provide early warning of potential non-compliance with ACGME accreditation requirements. All accredited programs (regardless of size) are required to participate in these surveys each academic year between the months of January and April.”).

